# The complete mitochondrial genome of the Chinese water snake *Myrrophis* (*Enhydris*) *chinensis* (Gray, 1842) (Reptilia: Homalopsidae)

**DOI:** 10.1080/23802359.2023.2248682

**Published:** 2023-08-24

**Authors:** Jun Ping, Zhiwang Xu, Yongpu Zhang

**Affiliations:** a Chengdu Institute of Biology, Chinese Academy of Sciences, Chengdu, China; b University of Chinese Academy of Sciences, Beijing, China; c College of Life and Environmental Science, Wenzhou University, Wenzhou, China

**Keywords:** *Myrrophis chinensis*, mitochondrial genome, next-generation sequencing

## Abstract

*Myrrophis* (*Enhydris*) *chinensis,* also known as the Chinese water snake, has been used for medicinal purposes, such as the treatment of ailments involving fever, headache, and joint pain. The complete mitochondrial genome of *M. chinensis* was assembled using next-generation sequencing. The mitochondrial genome was 17,302 bp in length and contained 37 genes, including 13 protein-coding, 22 transfer RNA (tRNA), 2 ribosomal RNA genes, and 2 non-coding control regions (D-loop). The light chain of replication origin was found between tRNA-Asn and tRNA-Cys in the WANCY gene cluster, which is consistent with published mitogenomes of Homalopsidae. The phylogenetic tree supported the monophyly of Homalopsidae species and implied that *M. chinensis* is the closest related species to *Myanophis thanlyinensis*. The mitochondrial genome of *M. chinensis* provides fundamental data for exploring mitochondrial genome evolution in snakes (Homalopsidae).

## Introduction

The Chinese water snake (*Myrrophis chinensis*) (Kumar et al. 2012) is an ovoviviparous colubrid snake widely distributed in Southern, Eastern, and Central China (including Anhui, Fujian, Guangdong, Guangxi, Hannan, Hunan, Hubei, Jiangsu, Jiangxi, Taiwan, and Zhejiang) (Zhao et al. 1998). *M. chinensis* is used in folk medicine to treat ailments such as fever, headache, and joint pain (Nóbrega et al. 2008). *M. chinensis* inhabits rice fields, ponds, and ditches that have important economic and scientific value. According to the ‘China Red Data Book of Endangered Animals’ and ‘China’s Red List of Biodiversity’, *M. chinensis* is considered a vulnerable species (Zhao et al. 1998; Wang et al. 2021). The assembly of *M. chinensis* mitogenome will facilitate research on species protection, delimitation, molecular evolution, and phylogenetic inference in snakes.

## Materials and methods

**Figure 1.**
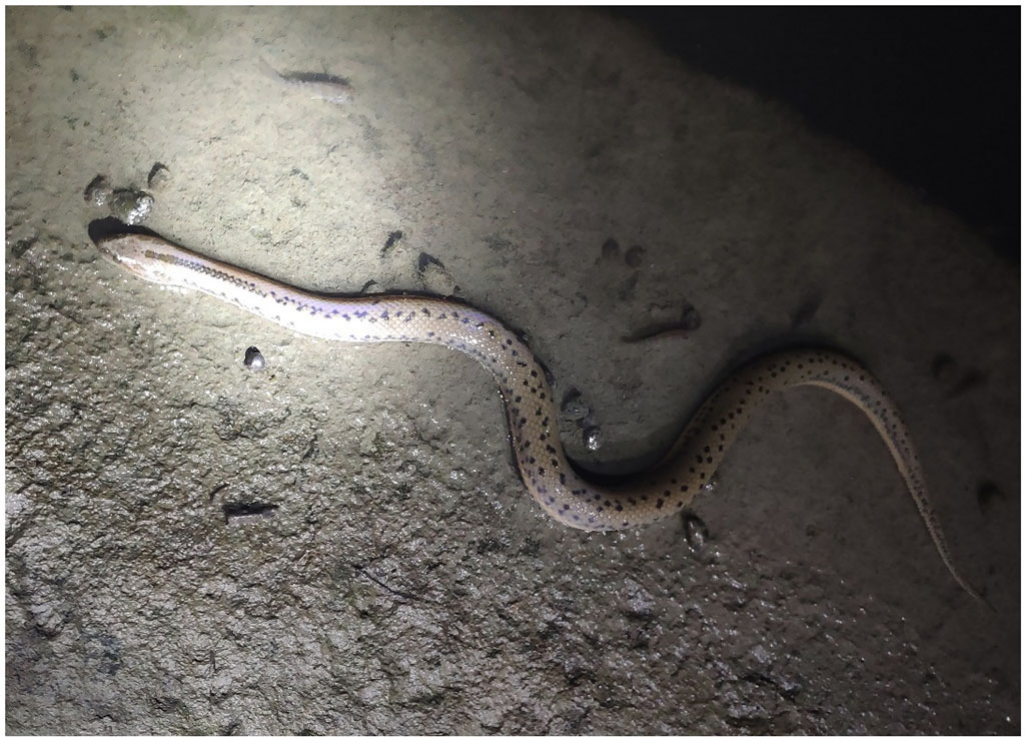
The specimen of *Myrrophis chinensis* from Quzhou City, Zhejiang Province, China (photo by Zhiwang Xu).

All procedures were approved by the Animal Care and Use Committee of the Wenzhou University (Permit Number: WZU-049). A male adult *M. chinensis* was collected from Quzhou City, Zhejiang Province, China, in August 2022 (N 119°2′39.89″, E 28°52′36.77″) (Figure 1). The muscle tissue was fixed with 95% ethanol and stored at −20 °C in the College of Life and Environmental Science, Wenzhou University (contact person: Yongpu Zhang, E-mail: zhangyp@wzu.edu.cn). The samples were transported to Genepioneer Biotechnologies Co. Ltd. (Nanjing, China) for genomic extraction and 150 bp paired-end library construction. Sequencing was performed using an Illumina NovaSeq 6000 sequencing platform (Illumina, USA). To verify the accuracy of the assembly, we aligned clean reads to the assembled genome to assess the depth of coverage. A total of 3220 reads were mapped to the complete genome sequence of *My. thanlyinensis* (NCBI GenBank ID: MW272554) in Bowtie 2 v2.2.4 (Langmead and Salzberg 2012), yielding 65 × coverage (supplemental Figure S1).

We used MITObim 1.9.1 (https://github.com/chrishah/MITObim) (Hahn et al. 2013) to assemble the *M. chinensis* mitochondrial genome using *My. thanlyinensis* as the reference. Additionally, a standard PCR method was used to amplify the mtDNA genes of *M. chinensis* for verification. Finally, we used tRNAscan-SE v.1.21 (http://lowelab.ucsc.edu/tRNAscan-SE) (Lowe and Chan 2016; Chan and Lowe 2019) and the MITOS web server (http://mitos2.bioinf.uni-leipzig.de/index.py) (Donath et al. 2019) to annotate the mitochondrial genome.

## Results

**Figure 2.**
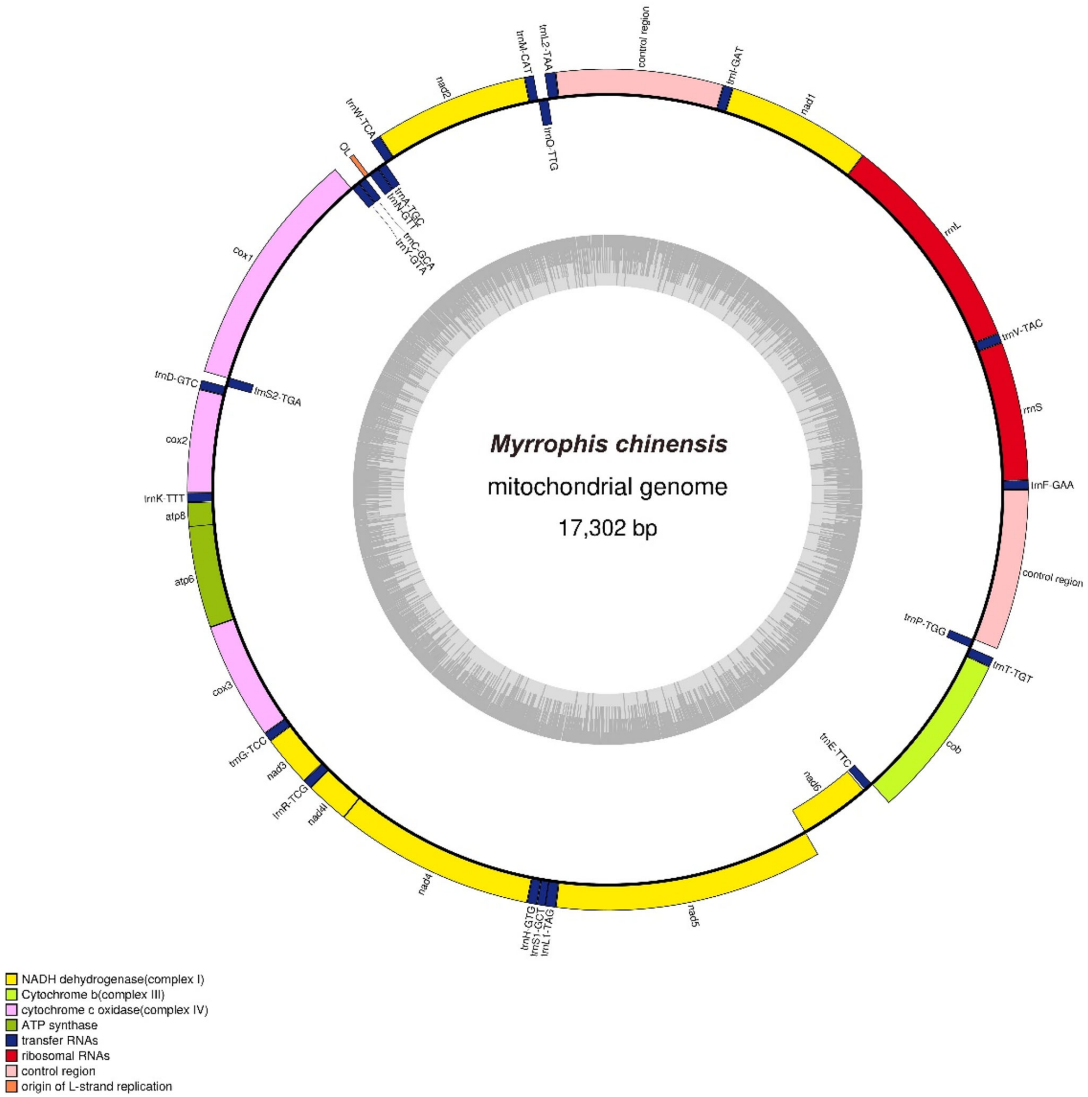
Gene map of the mitochondrial genome of *Myrrophis chinensis*. The forward orientation genes are located outside the circle, and the reverse orientation genes are located inside the circle. The inner gray circles represent GC content.

The mitochondrial genome of *M. chinensis* was 17,302 bp long (GenBank accession number: OP974098) (Figure 2) and consisted of 37 genes, including 22 transfer RNA (tRNA), 2 ribosomal RNA (16S and 12S), 13 protein-coding genes (PCGs), as well as 2 non-coding control regions (D-loop) and 1 light chain of replication origin (O_L_). Among the 13 PCGs, the longest and shortest were ND5 (1785 bp) and ATP8 (162 bp), respectively. The 12S and 16S rRNA genes were 922 and 1482 bp in length, respectively. One D-loop was located between tRNA-Ile and tRNA-Leu, with a length of 1111 bp, and the other was located between tRNA-Pro and tRNA-Phe, with a length of 1069 bp. The similarity between the two D-loops was 95.69%. The mitochondrial genome showed a relatively strong A and C bias, with base compositions of 33.23% A, 24.8% T, 28.7% C, and 13.26% G. Additionally, O_L_ was located between tRNA-Asn and tRNA-Cys in the WANCY tRNA cluster region, which is consistent with the mitochondrial genomes of two known Homalopsidae species, *My. thanlyinensis* and *Hypsiscopus* (*Enhydris*) *plumbea*.

**Figure 3.**
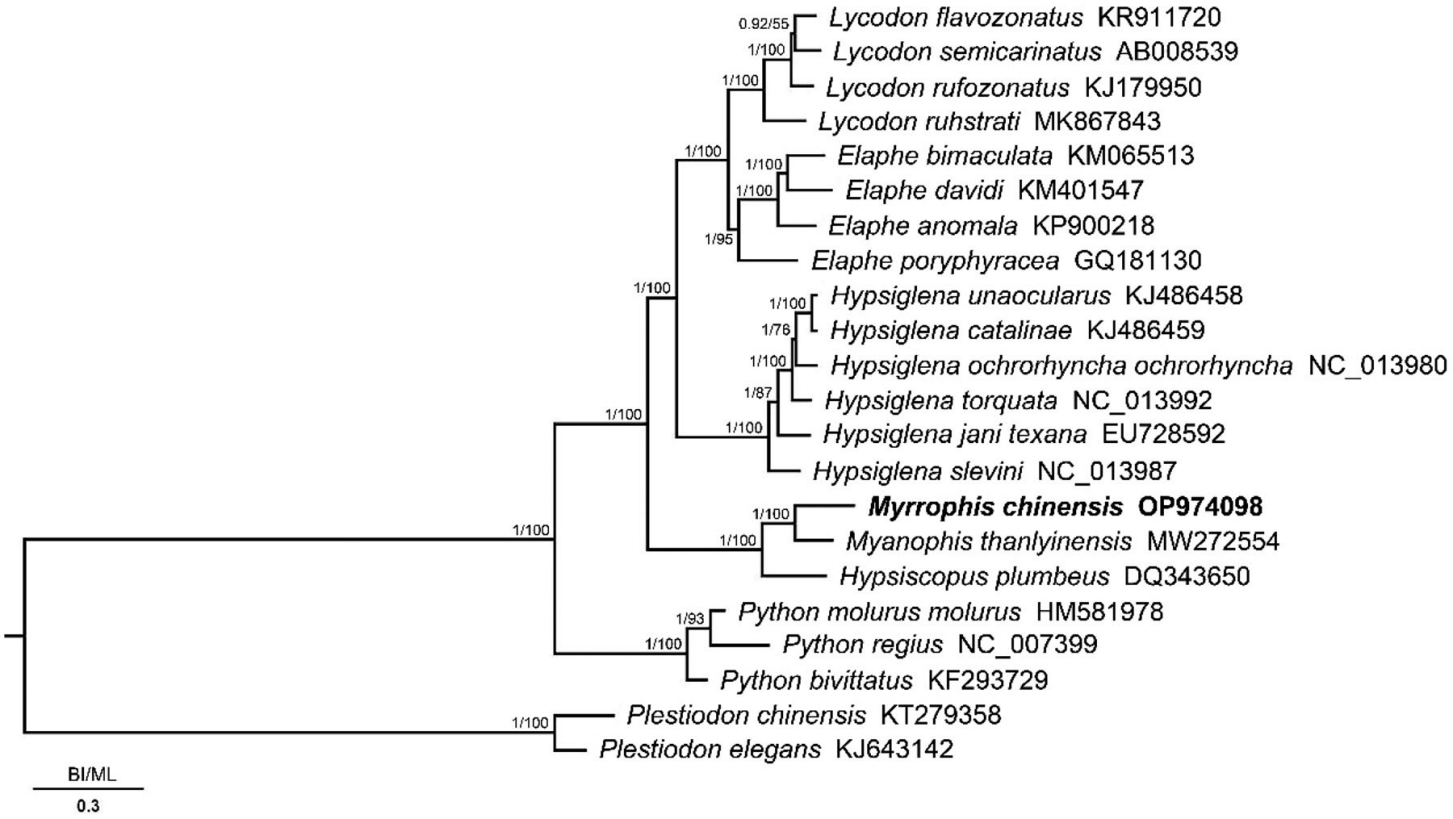
Phylogenetic analysis of the 13 protein-coding genes of *Myrrophis chinensis* and 21 closely related species based on Bayesian inference (BI) and maximum likelihood (ML). Node numbers show Bayesian posterior probabilities from BI and bootstrap percentages from ML. GenBank accession numbers are given with species names, and the phylogenetic placement of *M. chinensis* is highlighted in bold.

We constructed Bayesian inference (BI) implemented in MrBayes v.3.2.7a (Ronquist et al. 2012) and maximum likelihood (ML) implemented in RAxML v.8.2.12 (Stamatakis 2006) based on the 13 PCGs of *M. chinensis* and 21 related species to confirm the phylogenetic position of *M. chinensis.* The best-fitting substitution models and partitioning schemes were selected using PartitionFinder v.2.1.1 (Lanfear et al. 2017). Phylogenetic trees from the BI and ML analyses produced highly concordant topologies (Figure 3).

## Discussion and conclusion

In this study, we report the first complete mitogenome of *M. chinensis* established using high-throughput sequencing and assembly. The phylogenetic tree supported the monophyly of Homalopsidae, and *M. chinensis* showed the closest relationship to *My. thanlyinensis* (1.00 in BI and 100% in ML). The mitochondrial genome of *M. chinensis* provides fundamental data for exploring mitochondrial genome evolution in snakes (Homalopsidae).

## Supplementary Material

Supplemental Material

Supplemental Material

## Data Availability

Data supporting the findings of this study are available in the NCBI GenBank database (https://www.ncbi.nlm.nih.gov/) under accession no. OP974098. The associated BioProject, Sequence Read Archive (SRA), and Biosample numbers are PRJNA910348, SRR22586065, and SAMN32123642, respectively.
