# Supplementary figures and images for: The complete mitochondrial genome of the Chinese water snake Myrrophis (Enhydris) chinensis (Gray, 1842) (Reptilia: Homalopsidae)
Source: Mitochondrial DNA B Resour. 2023 Aug 24;8(8):899–902. doi: 10.1080/23802359.2023.2248682 (PMC10453999; doi:10.1080/23802359.2023.2248682)

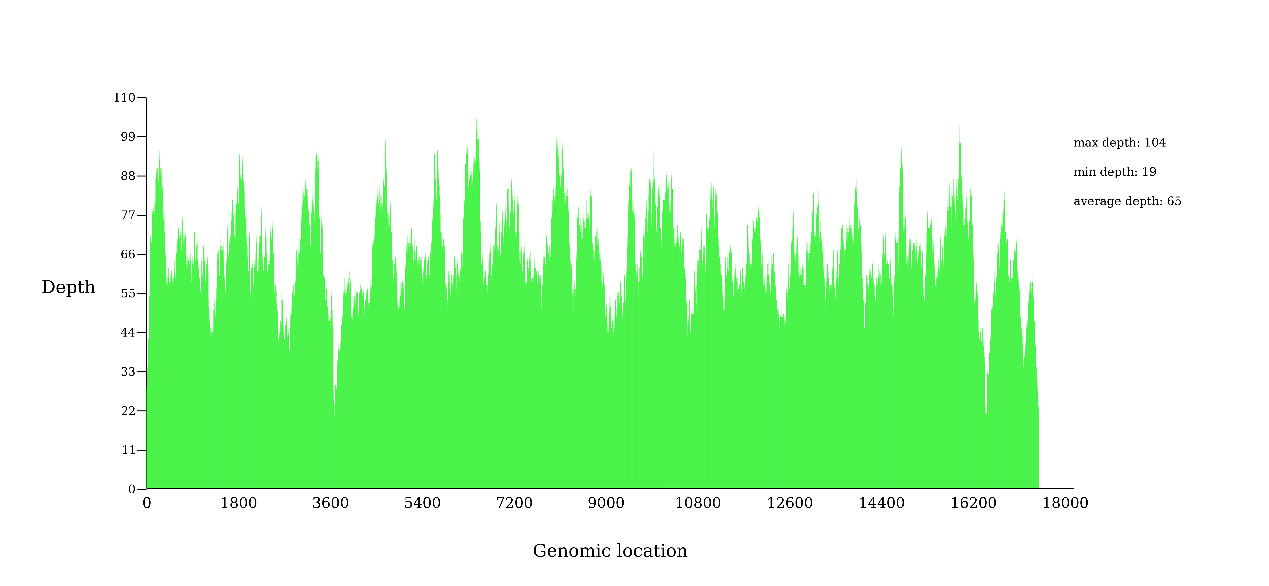


Figure S1. The coverage figure of the complete mitochondrial genome of *Myrrophis chinensis*.

Supplement: Supplemental Material [file TMDN_A_2248682_SM1454.docx]

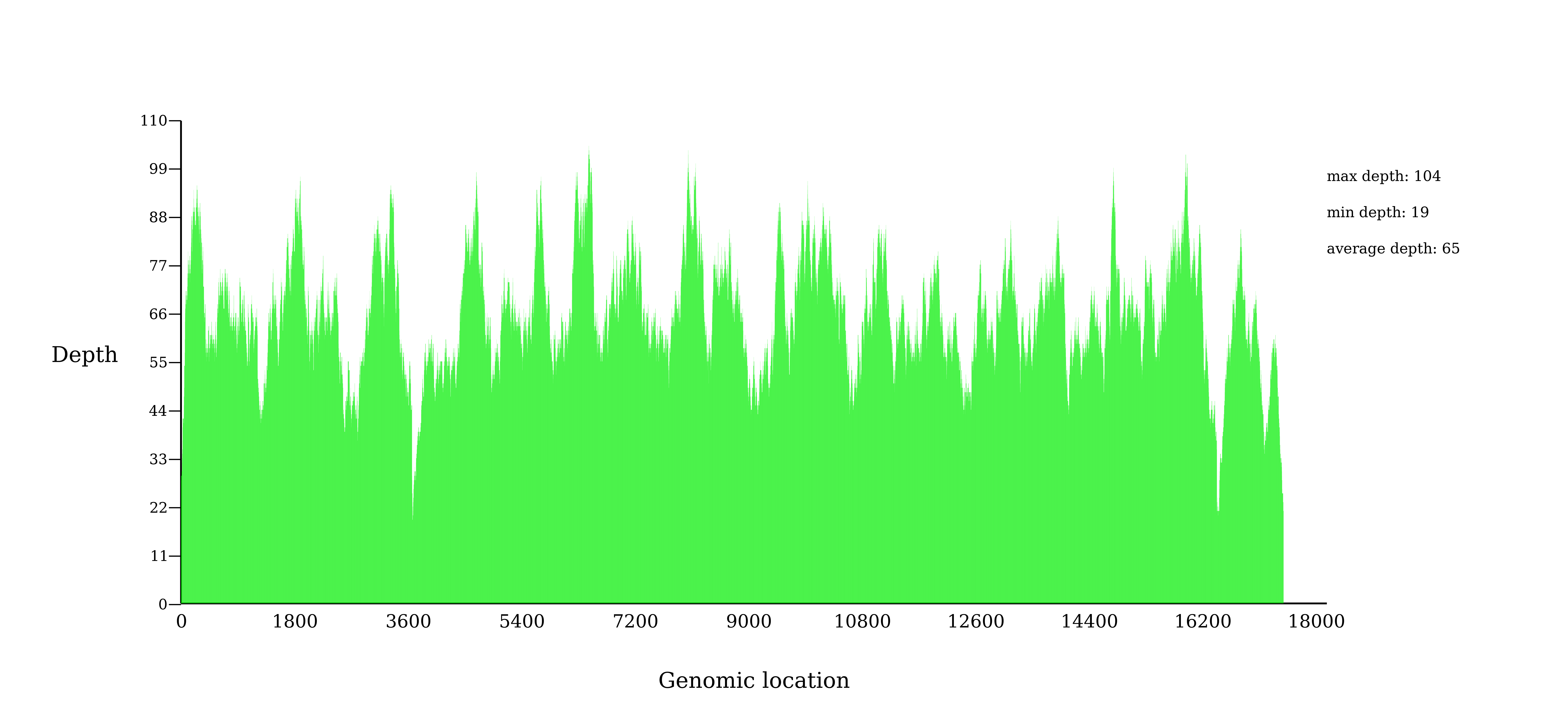

Supplement: Supplemental Material [file TMDN_A_2248682_SM1453.png]
